# A semiempirical method optimized for modeling proteins

**DOI:** 10.1007/s00894-023-05695-1

**Published:** 2023-08-22

**Authors:** James J. P. Stewart, Anna C. Stewart

**Affiliations:** Stewart Computational Chemistry, 15210 Paddington Circle, Colorado Springs, CO 80921 USA

**Keywords:** MOPAC, semiempirical, PM6-ORG, parameterization, proteins, reference data

## Abstract

**Context:**

In recent years, semiempirical methods such as PM6, PM6-D3H4, and PM7 have been increasingly used for modeling proteins, in particular enzymes. These methods were designed for more general use, and consequently were not optimized for studying proteins. Because of this, various specific errors have been found that could potentially cast doubt on the validity of these methods for modeling phenomena of biochemical interest such as enzyme catalytic mechanisms and protein-ligand interactions. To correct these and other errors, a new method specifically designed for use in organic and biochemical modeling has been developed.

**Methods:**

Two alterations were made to the procedures used in developing the earlier PMx methods. A minor change was made to the theoretical framework, which affected only the non-quantum theory interatomic interaction function, while the major change involved changing the training set for optimizing parameters, moving the focus to systems of biochemical significance. This involved both the selection of reference data and the weighting factors, i.e., the relative importance that the various data were given. As a result of this change of focus, the accuracy in prediction of heats of formation, hydrogen bonding, and geometric quantities relating to non-covalent interactions in proteins was improved significantly.

## Introduction

Semiempirical methods have been shown to be successful in modeling various phenomena that occur in enzymes. For example, the complete mechanism of the hydrolysis of a peptide bond by chymotrypsin was modeled [1], and the results were consistent with the accepted description of the catalytic cycle. This simulation involved locating, refining, and characterizing the various transition states between stable intermediates on the potential energy surface. In another study [2], the origin of the specificity of the nucleotide-pool sanitizing enzyme MTH1, an enzyme that can selectively hydrolyze oxidized nucleotides, was investigated. The interactions between the oxidized and non-oxidized nucleotides and the enzyme were modeled, and in 2013 a reason based on the individual residue contributions to the binding-site energies was proposed for the enzyme’s specificity. This explanation was essentially the same as a suggestion [3] involving specific promiscuity of an Asp-Asp recognition element proposed earlier in the same year.

Because the focus of semiempirical method development had been on modeling much smaller chemical systems, little or no effort had been expended in modeling non-interacting moieties, and, as a result, when validation tests for experimentally-determined protein geometries were applied to calculated geometries, a large number of unrealistic close contacts were found.

Experimentally, protein geometries of the type stored in the Protein Data Bank [4] (PDB) are obtained from a physical analysis of the system using techniques such as X-ray, neutron scattering, NMR, and, more recently, electron microscopy. In contrast, the computationally-optimized geometries of proteins are generated by energy minimization, typically starting from an experimentally-obtained geometry. Semiempirical computational methods are parameterized to reproduce chemical properties; therefore, although the resulting optimized geometries might be chemically acceptable, they might also be significantly different from the experimental geometries.

Experimentally-determined protein geometries are routinely analyzed for geometric anomalies, such as “clashes,” where pairs of atoms that are not covalently bonded and do not have a non-covalent stabilization interaction, such as hydrogen bonds, are closer together than would be expected. Clashes in proteins can be identified and quantified by programs such as Molprobity [5], which can generate a list of clashes and a simple scalar measure, called a “clashscore,” which indicates the quality of a structure.

Although clashes are useful in validating experimental geometries, from a computational chemical perspective, where energies dominate, clashes are of less importance because the energies involved are relatively small. Nevertheless, it is not sufficient that a computational model should be chemically realistic: it should also be physically realistic. Addressing this issue required that a small change be made to the semiempirical model.

## Background

### Covalent interactions

The earliest of the modern semiempirical Self-Consistent Field (SCF) methods, MNDO [6, 7], was published in 1977. That was the first time semiempirical methods had been able to predict the geometries of molecules and their heats of formation. MNDO was parameterized to reproduce the properties of systems where atoms were connected to other atoms by covalent bonds. That is, it was optimized for modeling molecules and polyatomic ions. MNDO was followed by more accurate methods, such as AM1 [8] in 1985, by PM3 [9, 10] in 1989, and by PM6 [11] in 2007. Each new method was built on the lessons learned from the previous method and attempted to correct existing faults, many of which were discovered only long after the method had been published.

### Non-covalent stabilizing interactions

These earlier methods did not adequately address the issue of non-covalent interactions, so hydrogen bonding and dispersion interactions were, for all practical purposes, non-existent. Two changes were made in 2013 to address this deficiency. A new method, PM7 [12], was developed that included dispersion and hydrogen bonding terms. Similar modifications were made to PM6, which consisted of adding energy stabilization functions, collectively called D3H4, and gave rise to two new methods: PM6-D3H4 [13] and PM6-D3H4X [14]. These developments allowed the range of systems that could be modeled with chemically-useful accuracy to be greatly expanded.

### Interactions that result in clashes

Although both PM6 with post-SCF corrections and PM7 were able to model enzymes and other proteins, justification for the further application of these methods was brought into question by the potentially serious problem mentioned earlier. Most proteins in the PDB, especially those deposited in recent years, have a low clashscore, but a survey of geometries of representative protein systems optimized using PM6-D3H4 and PM7 had an average clashscore over four times larger. These very high clashscores cast serious doubt on the level of confidence that could be placed on the predictions of these methods. Confidence in the computational model would be increased if changes could be made to the model so that the clashscores improved.

Analysis of the methods revealed that there was an absence of the weak, long-range, van der Waals (vdW) repulsive interaction. If present, such a repulsive interaction would be able to increase the interatomic separation between pairs of atoms that would not otherwise be attracted together, thereby reducing the incidence and severity of clashes.

### Protein – Ligand interactions

A potentially important application of semiempirical methods is the prediction of protein – ligand interaction (PLI) energies. In contrast to the small range of types of non-covalent interaction found in proteins, the range of possible non-covalent interactions between ligands and proteins is very large. As a result of individual non-covalent interactions between pairs of atoms, one on a ligand, the other on the protein, some other pairs of atoms are pulled inside their contact radii. In semiempirical methods, hydrogen bonding and electrostatic interactions that pull otherwise very weakly interacting pairs of atoms inside their contact radii are responsible for the unrealistic clashes observed in earlier methods. Interactions of this type are important in PLI energies, and only recently has access to data on such interactions become available.

## Methods

PM6-D3H4 was chosen as the starting point for the new method because it has been shown to be substantially more accurate [15] than PM7 in the modeling of non-covalent interactions in PLI complexes. PM6-D3H4X is similar to PM6-D3H4, the only difference being that the D3H4 correction is extended to include optimized parameters for the halogens. To avoid repetition, reference to PM6-D3H4 should be assumed to apply also to PM6-D3H4X, unless otherwise indicated.

### Modification of diatomic core-core interactions

Addressing the problem of adding a term to represent the repulsion of two otherwise non-interacting atoms in a protein requires an understanding of the environment of the atoms. Ignoring all other atoms, the force between them would be negligible until they approached the vdW contact distance, at which point a repulsive force would appear and increase as the interatomic distance decreased. In the absence of the other atoms, non-interacting atom pairs could not approach closer than the vdW contact distance, but, with current semiempirical methods, if the other atoms exerted an appropriate force on them, they could be pulled inside the vdW contact distance. An inspection of the Molprobity results showed that this fault could be corrected by the addition of a small repulsive force to the computational model for specific diatomic interactions. At vdW distances, all energy terms between pairs of atoms are very small, so any function that would produce the required repulsion force would only need to operate in that region. In PM6 the expression for the core-core repulsion energy between pairs of atoms is scaled using diatomic parameters [11], so, for simplicity, the value of the scaling factor, ***c***_***A,B***_, was modified by the addition of a term proposed recently [16], as shown in Equation 1.

1 $${{\boldsymbol{c}}_{\boldsymbol{A},\boldsymbol{B}}}^{\prime }={\boldsymbol{c}}_{\boldsymbol{A},\boldsymbol{B}}+\boldsymbol{a}.{\boldsymbol{e}}^{-\boldsymbol{b}{\left(\boldsymbol{c}-\boldsymbol{r}\right)}^{\textbf{2}}}$$

In this, ***a***, ***b***, and ***c*** are parameters which depend on the elements of the two atoms involved, and ***r*** is the interatomic distance. This function would be used at all interatomic distances greater than ***c*** Ångstroms; at smaller distances the additional term would be replaced simply by ***a***.

Because of its form, this function would have a negligible effect on the heat of formation but would exert a weak repulsive force on atoms in the region of their vdW contact distance.

### Parameters and Reference Data

Parameter optimization involves a training set of reference data. The original training sets used in developing PM6 and PM7 contained a large amount of data for systems that were not relevant to modeling biochemical systems, such as high-energy species such as difluoromethyl and the nitrogen dioxide cation. Many of these data were identified as being potentially detrimental to the accuracy of the current method, and consequently were removed from the training set.

Because the size of the parameter set was increased by the parameters in the repulsion functions, extra reference data had to be added to the training set, to allow these parameters to be defined. Each of these data was designed to represent one and only one diatomic vdW repulsion. Conventional reference data from experiment or from high-level calculations were unsuitable for this task; instead, small chemical systems that were proxies for each diatomic interaction were used. Each repulsion interaction was represented by two systems: one consisting of two small molecules separated by a large distance, typically 50 Å, and the other consisting of the same two molecules separated by the vdW contact distance. The difference in energy of these systems then formed a proxy for the vdW repulsion.

In practice, clashes involved only a small number of diatomic interactions and these were restricted to the elements H, C, N, O, and S. Examples of these are listed in Table 1, along with the pair of molecules that represented each interaction. Molecules were chosen and oriented so that the specific pair of atoms of interest, one in each molecule, were nearest to each other and all other atom pairs were significantly further away. This arrangement allowed each diatomic interaction to be represented by a single proxy.

**Table 1:** Examples of atom pairs involved in clashes

Pairs of non-interacting atoms	Proxy systems in training data-set
H - H	H_2_ – H_2_
C - H	HNC – H_2_
C - O	HNC – CO_2_
O - H	CO_2_ – H_2_
O - N	NH_3_ – CO_2_
S - O	H_2_S – CO_2_

### Non-covalent interactions

The energy of the non-covalent interaction between a ligand docked to the binding site of an enzyme is a useful measure of the binding efficiency of that ligand, and, by inference, the effectiveness of the pharmacophore. One estimate of the accuracy of prediction of individual non-covalent interactions can be obtained by comparing the calculated binding energy and reference binding energies.

Non-covalently bound ligands are stabilized by electrostatic, hydrogen bonding, and dispersion terms. However, in addition to these terms, there are other protein-ligand interactions that are destabilizing. This occurs when various stabilizing non-covalent interactions cause a ligand to approach a protein so closely that other pairs of ligand and protein atoms, which otherwise would not interact, start to repel each other. The absence of these destabilizing interactions has been reported [17] to give rise to severe errors when either PM7 or PM6-D3H4 was used in modeling repulsive contacts

Correcting this deficiency was straightforward. Using CCSD(T)/CBS methods, a benchmark data set of repulsive interaction energies, named R739x5, was developed [17]. Entries in the R739x5 set were then used in the construction of two data-sets: a small training set for use in parameter optimization and a much larger survey set for determining the accuracy of prediction of these interactions.

For both non-covalent and repulsive interactions each reference datum involved three species. One consisted of the complex of the two species involved; for non-covalent interactions, these were two species in their equilibrium geometry, and for the repulsive interactions, as recommended [17], these were positioned on the repulsive part of the potential energy surface at about 2 kcal mol^-1^ above the well-separated components. The other two moieties were the individual species, either calculated individually or as a well-separated pair. The resulting computed heats of formation were then used for calculating the interaction energy.

### Weighting Reference Data

Before individual reference data can be used in optimizing the values of parameters, they must first be rendered dimensionless. Prior to the development of PM6, only data relating to individual species were used, and the weighting factors for heats of formation spanned a small range, up to a maximum of about 1.0/(kcal mol^-1^). During the parameter optimization of PM6, reference data representing hydrogen bonds were introduced. These involved systems where non-covalent interatomic separations spanned the range from 1.5 to 2.0 Ångstroms, in contrast to the normal O-H and N-H covalent bond lengths of about 1.0 Ångstroms, and the energies involved were only a small fraction of those involved in covalent interactions. In order to compensate for the difference in magnitude, the weighting factor for hydrogen bond energies was increased to about 10/(kcal mol^-1^).

A second set, consisting of data that represents Molprobity clashes, will now be introduced. Molprobity clashes involve pairs of atoms that are not involved in even the weak non-covalent interaction of the type found in hydrogen bonding, and the interatomic separations are typically in the range 2.5 to 3 Ångstroms. At such large distances, the forces acting on the atoms are minute compared even to those involved in hydrogen bonds.

Each type of diatomic clash was represented by precisely one Clash datum in the training set. In the original data set there would be a large number of similar diatomic pairs separated by distances approximately the same as that in the Clash datum, but in contrast with the Clash datum, these would, by definition, be connected covalently by one or more atoms. Because of the large disparity in the number of data in the original set, the weighting factor for systems in the second set had to be increased significantly. This, together with the increase needed to compensate for the very small energies involved at such large distances, made it necessary to increase the weighting factor for Clash data to between 30/(kcal mol^-1^) and 100/(kcal mol^-1^).

### Parameter optimization

All parameters for the elements H, C, N, O, F, P, S, Cl, Br, and I were optimized simultaneously. In the first cycle, all proxy energies were set to zero; this meant that the vdW repulsion term was not represented in the training set. The resulting parameters were then used in performing unconstrained geometry optimizations on a few proteins. As expected, the calculated optimized geometries had a large clashscore. The largest clashes in the optimized geometries were then used as a guide to update the weights of the corresponding reference proxy data for the next cycle of parameter optimization. This sequence was repeated until the average clashscore became acceptably small.

Eight other elements commonly found in enzymes, Na, Mg, K, Ca, Fe, Co, Zn, and Se, were then parameterized. They had not been included in the original parameterization because the accuracy of the available reference data for small molecules involving any of these elements was too low, and also because they were unlikely to be involved in clashes. In contrast to the common organic elements, these parameter optimizations were relatively simple in that only covalent or strong ionic interactions were important, and, of these, only interactions with H, C, N, O, and S were relevant. In this set of optimizations, all the parameters for the elements previously parameterized were held constant.

When the D3H4 correction was made to PM6, no changes were made to the original PM6 parameters. The D3H4 correction was simply “added on” to the results of a normal PM6 calculation, so, although the resulting method was more accurate in predicting non-covalent interaction energies, the new heats of formation could no longer be related to the reference data values. This limitation would, of course, not be relevant when modeling PLI geometries and interactions, but would affect heats of formation of intermediates particularly when modeling enzyme-catalyzed reaction mechanisms. In the current work all the parameters in PM6-ORG, i.e., the PM6, D3H4, and vdW parameters, were optimized simultaneously, therefore the predicted heats of formation could be related to the reference data values.

The new PM6-ORG method has been added to MOPAC [18], and can be activated by keyword “PM6-ORG.”

## Results

### Small molecules

A comparison of PM6-ORG with PM6-D3H4 and PM7 is shown in Table 2. Errors in heats of formation and bond angles decreased somewhat. A problem occurred in predicting average errors in bond-lengths, where PM6-D3H4X predicted some bonds involving oxygen covalently bonded to chlorine, for example in perchloric acid, to be unrealistically long. To ensure that the Average Unsigned Error (AUE) in bond-lengths was meaningful, only those errors that were less than one Ångstrom were used in calculating the AUE. Also, because both PM6-D3H4 and PM6-D3H4X were optimized to model non-covalent interactions, the AUE in heats of formation predicted by these methods were very large, 10.50 and 12.59 kcal mol^-1^, respectively. For this reason, the reported value of the AUE of heats of formation shown in the table is for PM6, not PM6-D3H4.

**Table 2:** Average unsigned errors in small molecules

Quantity	Units	No. in set	PM6-ORG	PM6-D3H4	PM7
∆H_f_	kcal mol^-1^	1690	3.88	4.77^a^	4.22
Dipole	Debye	128	0.37	0.38	0.47
I.P.	Electron Volts	216	0.55	0.48	0.47
Bond lengths	Ångstroms	492	0.017	0.022	0.019
Angles	Degrees	223	2.59	3.18	2.96

^a^:Average unsigned error for PM6; the AUE for PM6-D3H4 is not valid (see text)

### Non-covalent and repulsive interactions

Average unsigned errors for five sets of systems composed of pairs of molecules, one set involving ions, and one set involving only repulsive interactions are presented in Table 3.

**Table 3:** Average unsigned errors for various sets of interacting pairs of molecules and ions (kcal mol^-1^)

Data-set	No. in set	PM6-ORG	PM6-D3H4	PM7
S22 [19]	22	0.87	0.65	0.76
S12L [19]	12	8.47	10.62	23.63
S66 [19]	66	1.00	0.49	0.77
L7 [20]	7	5.38	3.92	6.41
X40 [19]	40	1.15	1.19	1.83
Ionic H-bonds [19]	15	1.39	1.35	1.44
Repulsive contacts [17]	526	1.70	1.87	2.44
All interactions	688	1.73	1.82	2.58

### Distribution of errors in Repulsive Interactions

**Fig. 1:**
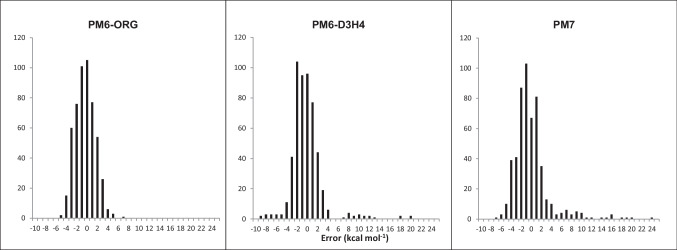
Histogram of errors in Repulsive Interactions

Average unsigned errors for these species decreased to 1.70 kcal mol^-1^ from 1.87 for PM6-D3H4 and 2.44 for PM7. More important, the kurtoses of the error distribution for the repulsive interactions changed significantly. As shown in Figure 1, the kurtosis of the PM6-ORG distribution of errors was -0.1, i.e., essentially normal, whereas both PM6-D3H4 and PM7 were strongly leptokurtic, with kurtoses of 12.6 and 10.2, respectively.

### Proteins

All protein calculations were performed using the MOPAC [18] program. A set of 21 proteins, shown in Table 4, was used in testing. Each system modeled was based on a PDB geometry downloaded from the RCSB PDB [21].

**Table 4:** Set of Proteins used in the survey

Protein	PDB ID	Resolution (Å)	Year	No. of residues	No. of atoms	Net charge
Crambin	4FC1	1.10	2012	46	768	0
Acyl-Coenzyme A	7DES	1.45	2022	96	1745	-1
Zinc finger domain of KLF4	2WBS	1.70	2009	87	1787	+7
Barnase	1A2P	1.50	1999	108	2102	+2
Zinc endoprotease	1C7K	1.00	2000	132	2279	+1
Apoptosis inhibitor	7PDJ	4.20	2022	166	2496	-9
Human hemoglobin, chain A	5WOH	1.58	2018	137	2723	-1
Flavodoxin	5WID	1.68	2019	144	2858	-3
Rab6	1D5C	2.30	2000	162	2858	0
Adenylyltransferase	1O6B	2.20	2003	169	2904	+2
Calcium binding domain	1UOW	1.04	2003	157	3066	+9
Magnesium loaded ALG-2	5JJG	1.72	2016	168	3088	+1
Peridinin-chlorophyll	2X20	1.95	2010	151	3537	0
Green Fluorescent Protein	5WJ2	2.41	2018	235	3874	-5
Bacteriorhodopsin	5ZIM	1.25	2018	228	4200	0
Chymotrypsin	5J4Q	2.30	2017	305	4684	+22
3CLpro	7JUN	2.30	2020	306	4879	-5
Potassium channel	1JVM	2.80	2001	393	5752	+7
P450	7TTP	1.80	2022	345	6093	-14
Sodium channel	4CBC	2.66	2014	372	6345	-1
Transcobalamin	2V3N	2.73	2007	405	7128	+9

Proteins were selected with the objective of representing as wide a range as possible. Thus 1JVM and 4CBC contain isolated alkali metal ions; 1UOW contains calcium, 1C7K and 2WBS contain zinc covalently bound to nitrogen, oxygen, and sulfur; 2V3N contains cobalt in a corrin ring; 2X20 contains magnesium in a porphyrin ring and 5JJG contains magnesium octahedrally coordinated; 7TTP and 5WOH both contain iron in a heme ring. 7JUN is a 3-chymotrypsin-like cysteine protease, and 5J4Q is chymotrypsin, a serine protease; the barrel protein 5WJ2 consists mainly of beta sheet protein, and 5ZIM consists mainly of alpha helices.

Only minimal editing was done in preparing proteins for modeling. In general, this involved using keywords in MOPAC to add hydrogen atoms and resolve bonding ambiguities, primarily to define which Lewis structure should be used in complicated ring systems of the type that occur in chlorophyll, heme, and corrin.

All water molecules were retained and bulk solvent was represented by the implicit solvation method COSMO [22]. In general, all commonly ionized residues were ionized. The MOZYME [23] localized molecular orbital (LMO) method was used in determining the net charge on each system, and in solving the SCF equations. To reduce the computational effort, a cutoff of 6 Ångstroms was used for the NDDO [24] approximation. This resulted in a significant increase in speed with only a small change in the calculated heat of formation, and a negligible difference in the optimized geometry.

Geometry optimization was performed using the L-BFGS [25, 26] method, which has proven to be a highly efficient method for locating a minimum-energy geometry in complicated systems such as proteins. However, for modeling very low-energy phenomenon of the type being considered here, the default settings for the L-BFGS method had to be adjusted to increase the precision. To do this, the optimization was continued until no further decrease in the heat of formation could be achieved after 60 cycles of optimization. Although an accurate estimate of the precision could not be made, based on the convergence properties of the optimization, a rough estimate was that the final heat of formation was within 1 kcal mol^-1^ of the minimum.

### Molprobity analysis

Analyses of the optimized structures were performed using Molprobity [5, 27]. This program identified atom pairs whose interatomic separation would be expected to be influenced mainly by their vdW stabilization. All distances that were shorter by 0.4 Å or more than that which was expected were identified as clashes. Overlaps of this magnitude “…cannot occur in the actual molecule, but mean that at least one of the two atoms is modeled incorrectly” [27].

Molprobity was developed primarily for structure validation of protein structures resulting from physical analyses. PDB files often do not include hydrogen atoms, but their presence is essential for structure validation, so, when Molprobity starts, the normal procedure is for any missing hydrogen atoms to be added to generate a standard starting configuration.

In this work, the structures to be validated are the predicted geometries of semiempirical computational chemistry methods. An essential prerequisite of these methods is that they must be as realistic as possible; therefore hydrogen atoms must be present to satisfy valence and chemical requirements. All the residues that would likely be ionized, i.e., aspartic and glutamic acids, the bases lysine and arginine, and the amino and carboxyl chain termini, were ionized. This operation had to be performed before the geometry of the system could be optimized.

These two types of model are sufficiently different as to justify altering the analysis process normally used in Molprobity. To allow a fair comparison to be made of the reported and calculated geometries, the Molprobity “Add hydrogens” option was not used. Instead, the original PDB structures were hydrogenated and the positions of the hydrogen atoms optimized using PM6-ORG. All further mention to PDB structures should be understood as referring to the original PDB geometry with hydrogen atoms added and their positions optimized in the same manner as in the other geometry optimizations.

Clashscores for the original geometry and for the geometries predicted by semiempirical methods are shown in Table 5.

**Table 5:** Comparison of protein Molprobity clashscores for different methods

Protein	PDB(a)	PDB(b)	PM6-ORG	PM6-D3H4	PM7
Crambin	0	0.00	6.23	28.04	21.81
Acyl-Coenzyme A	4	5.40	6.08	27.01	20.93
Zinc finger domain of KLF4	2	2.15	5.74	38.74	29.41
Barnase	2	1.18	7.06	32.94	27.06
Zinc endoprotease	2	1.04	8.80	41.95	27.96
Apoptosis inhibitor	•••	7.61	2.00	26.04	26.44
Human hemoglobin, chain A	5	4.22	2.81	33.30	26.27
Flavodoxin	3	4.75	3.89	39.31	31.53
Rab6	9	9.73	3.74	31.45	31.07
Adenylyltransferase	14	12.83	4.29	41.60	31.88
Calcium binding domain	10	11.91	5.16	42.49	36.54
Magnesium loaded ALG-2	1	2.17	3.97	27.43	17.32
Peridinin-chlorophyll	10	8.14	5.43	33.23	31.88
Green Fluorescent Protein	3	9.47	5.96	38.17	34.38
Bacteriorhodopsin	14	12.64	2.67	23.58	29.42
Chymotrypsin	10	9.21	5.61	34.83	28.31
3CLpro	1	2.35	5.77	30.34	28.20
Potassium channel	37	25.92	2.96	23.83	29.40
P450	2	3.12	5.70	33.81	27.38
Sodium channel	8	14.81	0.00	24.53	32.92
Transcobalamin	5	4.69	5.30	28.00	33.91

a:Values from the Protein Data Bank validation report

b:Values from this work

### Comparison of outliers in bond-lengths and angles

Molprobity was also used in analyzing the geometries for possible faults in bond-lengths and angles. Two outliers that occurred most frequently involved the bond-lengths for histidine Nδ1 – Cε1 and arginine Nε – Cζ both of which were about 0.04 Å, or 3%, too large. Of the angles, the most common outlier involved residues that had the trio of atoms Cα–Cβ–Cγ, where the calculated angle was 5 – 8 degrees too small.

### Comparison of overall geometries

For each system the root mean square deviation (RMSD) between calculated and X-ray backbone geometries was calculated; this provided a useful scalar measure of the difference between the geometries of two systems. Only atoms with the PDB label N, CA, or C in each system were used, and the geometry of the calculated system was rigidly rotated and translated as required to achieve the RMSD. Individual RMSD’s are shown in Table 6.

**Table 6:** Root-mean–square deviation between calculated and X-ray geometries of protein backbones (Å)

Protein	PM6-ORG	PM6-D3H4	PM7
Crambin	0.797	0.537	0.526
Acyl-Coenzyme A	0.771	0.649	0.605
Zinc finger domain of KLF4	0.816	1.215	1.117
Barnase	0.857	0.669	0.740
Zinc endoprotease	0.990	0.742	0.741
Apoptosis inhibitor	1.033	1.080	1.409
Human hemoglobin, chain A	0.686	0.588	0.736
Flavodoxin	0.637	0.587	0.681
Rab6	0.863	0.788	0.802
Adenylyltransferase	1.106	0.918	1.075
Calcium binding domain	0.821	0.634	0.808
Magnesium loaded ALG-2	0.879	0.802	0.809
Peridinin-chlorophyll	0.825	0.736	0.959
Bacteriorhodopsin	0.934	0.958	1.045
Green Fluorescent Protein	0.962	0.642	0.801
Chymotrypsin	0.951	0.970	1.168
3CLpro	1.108	0.910	1.224
Potassium channel	1.765	1.036	1.146
P450	1.121	0.933	1.040
Sodium channel	1.220	0.997	1.736
Transcobalamin	1.094	1.029	1.113

### Comparison of volumes

Given that most proteins are highly compact macromolecules [28] and that clashes are the result of otherwise non-interacting atoms becoming too close, if a protein chain became less compact, by increasing its inter- and intra-chain distances, the incidence of clashes could be expected to drop. This would, however, represent a trivial and unrealistic way to reduce the frequency of clashes. A simple measure of how compact a protein is, can be inferred from its volume. In the COSMO implicit solvation method used here, the Solvent Accessible Surface (SAS) is a well-characterized quantity [22] which can be used in evaluating the volume of a system. A comparison of the volumes inside the SAS for the experimental and calculated structures is presented in Table 7.

**Table 7:** Volumes of protein systems

Protein	Volume (Å^3^)				Volume percent change		
	PDB	PM6-ORG	PM6-D3H4	PM7	PM6-ORG	PM6-D3H4	PM7
Crambin	6786	6315	6353	6308	-6.95	-6.38	-7.04
Acyl-Coenzyme A	15285	14218	14561	14360	-6.98	-4.74	-6.05
Zinc finger domain of KLF4	15048	14547	14575	14230	-3.33	-3.15	-5.44
Barnase	18241	16778	17323	16625	-8.02	-5.03	-8.86
Zinc endoprotease	20168	18400	18666	18231	-8.77	-7.45	-9.61
Apoptosis inhibitor	22983	21646	21360	20600	-5.82	-7.06	-10.37
Human hemoglobin, chain A	23364	22094	21164	21364	-5.44	-9.42	-8.56
Flavodoxin	24405	22733	22517	22133	-6.85	-7.74	-9.31
Rab6	24754	23104	23837	23221	-6.66	-3.70	-6.19
Adenylyltransferase	25493	25389	24945	24128	-0.41	-2.15	-5.35
Calcium binding domain	25911	24313	25168	24037	-6.17	-2.87	-7.23
Magnesium loaded ALG-2	26510	25240	25618	25173	-4.79	-3.36	-5.04
Peridinin-chlorophyll	29872	28131	28381	27120	-5.83	-4.99	-9.21
Bacteriorhodopsin	35478	32633	33304	32611	-8.02	-6.13	-8.08
Green Fluorescent Protein	33660	31558	32377	31489	-6.24	-3.81	-6.45
Chymotrypsin	40672	38941	39270	38236	-4.26	-3.45	-5.99
3CLpro	42947	39716	40856	39444	-7.52	-4.87	-8.16
Potassium channel	49257	46880	47054	45786	-4.83	-4.47	-7.05
P450	52059	48486	48745	47287	-6.86	-6.37	-9.17
Sodium channel	53356	53313	50913	49213	-0.08	-4.58	-7.76
Transcobalamin	62042	57107	58339	56699	-7.95	-5.97	-8.61

## Discussion

Proteins present a unique problem in that they are too large to be used in conventional parameter optimization operations. This operation uses reference data for a large number of small systems, so the properties for each datum can be calculated rapidly, allowing a complete parameter optimization to be carried out in only one or two days using a 3GHz computer. However, calculation of even one datum for an entire protein system would require more computational effort than that for all the other systems combined, so simply adding proteins to the optimization was impractical.

An alternative, using a single proxy reference datum to represent each particular type of clash, eliminated the need for any proteins to be present in the optimization.

A consequence of this use of a single proxy datum to define three new parameters was that these parameters would not be uniquely defined, an ambiguity that was resolved by the presence of the large amount of reference data on small systems. Their presence in the optimization automatically had an effect on the new parameters, allowing them to become uniquely defined.

Optimizing parameters for use in modeling proteins has hitherto been impractical because of the very large computational effort that would be required. However, in the approach described here, by constructing proxy reference data that represent individual interactions of the type that occur in proteins but do not occur in small chemical systems, a considerable reduction in time has been achieved. When these proxy reference data representing long-range interactions are used together with enough reference data on small systems to allow short-range chemical properties to be properly represented, optimization of parameters for use in modeling proteins requires approximately the same time as that for a conventional optimization.

**Fig. 2:**
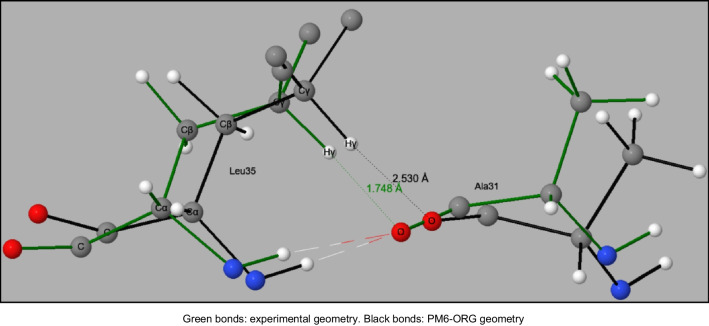
Largest clash in PDB geometry for the Potassium Channel, 1JVM

A comparison was made of individual clashes in original PDB structures and their equivalent in PM6-ORG optimized structures. As expected, all the results from geometry optimization were consistent with the assumption that the largest changes would involve the most flexible coordinates. This can be illustrated using the potassium channel protein 1JMV, the protein whose experimental geometry had the largest clashscore. Within this system, the largest clash involved Ala31 and Leu35 of chain A, as shown in Figure 2, together with the optimized PM6-ORG geometry. An inspection of the leucine side-chain showed that the large increase in O–Hγ distance, 0.78 Å, was being caused mainly by a rotation of the C-Cα-Cβ-Cγ torsion angle.

A deficiency in two semiempirical methods, PM7 and PM6-D3H4, which had allowed pairs of atoms in proteins to approach closer than expected, has been corrected by the addition of a simple empirical function that represented the vdW repulsion, a term that had not been important when only small systems were being modeled. After this correction was made, the clashscores decreased to less than 20% of their values when PM7 and PM6-D3H4 were used, and to 60% of the original PDB clashscore.

Previous semiempirical methods modeled chemical systems by using two types of interatomic interaction: those that represented strong covalent interactions, such as chemical bonds, and the much weaker non-covalent stabilization interaction caused by hydrogen bonds, electrostatics, and dispersion effects. The addition of the vdW repulsion interaction represents a new and third type of term in NDDO semiempirical methods. Although this term is the smallest and weakest of the three interactions, its absence from the computational model was the direct cause of large geometric errors in the predicted structures of proteins.

Optimizing semiempirical models to more accurately reproduce properties of proteins was achieved by the development of reference data that acted as proxies for individual diatomic interactions in proteins. This strategy is highly flexible, and would likely be applicable to addressing other errors found while modeling biochemical systems.

### Comparison of methods for modeling small molecules

Compared with PM7, PM6-ORG represents a reduction of 8% in AUE in predicted heats of formation, a 21% reduction in AUE in dipole moments, a 10% reduction in AUE in bond-lengths, and a 12% reduction in AUE in bond-angles. One metric, the ionization potential, showed an increase of 17%.

As noted earlier, because the D3H4 correction was added to the un-modified PM6, the heats of formation predicted by PM6-D3H4 were shifted by more than 10 kcal mol^-1^, on average, and would have rendered any comparison of AUE meaningless. This particular fault was corrected in the current parameterization.

### Comparison of methods for modeling intermolecular interactions

The most dramatic change was in the repulsive contacts, where the range of errors decreased considerably, from about 30 kcal mol^-1^ for PM6-D3H4 and PM7, to about 12 kcal mol^-1^ for PM6-ORG. Because the range of errors was significantly reduced, the incidence of large errors in non-covalent interactions would also be reduced. This would be expected to result in a corresponding improvement in the accuracy of the prediction of protein - ligand interaction energies, particularly those involving unusual combinations of elements.

### Comparison of methods for modeling proteins

Average errors for clashscores, backbone deviation, and percent volume change are shown in Table 8.

**Table 8:** Average errors in Proteins

Quantity	PDB	PM6-ORG	PM6-D3H4	PM7
Clashscore	7.48	4.72	32.41	28.76
RMS deviation (Å)		0.96	0.83	0.97
Volume (% change)		-5.80	-5.13	-7.60

The largest reduction in errors occurred in the clashscores, which implies that the computational model is more realistic and should help alleviate some of the doubts regarding the usefulness of these methods for modeling protein behavior. Both the RMSD and the volume change are intermediate between PM6-D3H4 and PM7, although the RMSD for PM6-ORG was significantly worse than that for PM6-D3H4. This deterioration could be attributed to the possible presence of large distortions in the X-ray structures of the sodium and potassium channel proteins, as implied by their reported unusually large PDB clashscores. When these two proteins were removed from the RMSD calculation, the PM6-OPT value dropped to 0.91 Å.

### Hydrogen bond lengths

**Fig. 3:**
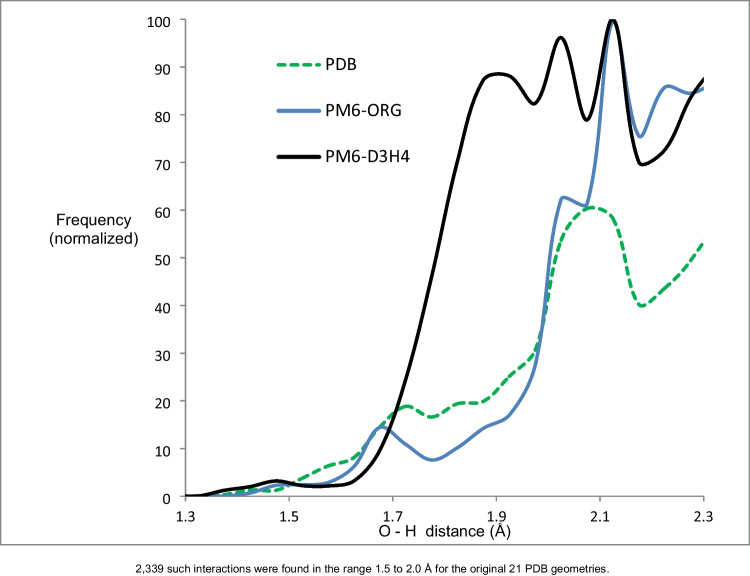
Relative frequency of non-covalent oxygen - hydrogen interactions

An anomaly was found in the PM6-D3H4 distribution of hydrogen-bond lengths involving two oxygen atoms in the region between 1.7 and 2.1 Å, in that the number of hydrogen bonds predicted using PM6-D3H4 was significantly larger than that predicted using the original PDB geometry, as shown in Figure 3. Examination of the types of hydrogen bonds showed that there was an increased propensity for water molecules to form hydrogen-bonds, for example, in chymotrypsin, H_2_O306 formed two hydrogen bonds, both of length 2.01 Å, with the peptide oxygen of Ala111. A survey was run to determine the frequency of a water molecule forming two hydrogen bonds with the same oxygen atom. No examples were found when the PDB or PM6-ORG geometry was used, but 35 examples were found when PM6-D3H4 was used.

### Proteins

Although the objective of the new method is to improve the ability to model enzyme mechanisms and other dynamic phenomena that occur in proteins, any investigation into such phenomena would, because of their great complexity, require considerable effort. Therefore, for the purposes of this report, only static properties were examined.

### Individual proteins

Bacteriorhodopsin is a good example of a protein that contains a large amount of α-helix. Its structure consists of a stack of seven α-helices surrounding a protonated Schiff base formed by Nξ on Lys216 and a retinal group. This Schiff base forms [29] strong hydrogen bonds with Asp85 and H_2_O402, and in turn the H_2_O402 moiety forms strong hydrogen bonds with Asp85 and Asp212.

All these features, i.e., the helices, the Schiff base and the compact hydrogen bonded structure, were reproduced by PM6-ORG. In both the original PDB structure and in the optimized PM6-ORG structure the charge distribution in the Schiff base was delocalized over the extended conjugated π-system of the retinal. This prediction is consistent with a recent report [30] on related rhodopsins, where, as the authors of that paper noted, it is in variance with the current consensus opinion that the positive charge would be localized at the site of the Schiff base.

Green fluorescent protein (GFP) provides a good example of the other main secondary structure in proteins: the anti-parallel β-sheet. It consists of a single protein chain folded into 11 β-strands that form a barrel. Inside the barrel is a chromophore of the type *p*-hydroxybenzylidene-imidazolidone composed from residues Ser65, Tyr66, and Gly67. This chromophore is held in place by a strong electrostatic interaction between the ionized guanidine group on Arg96 and the O2 on the imidazole ring. Like the chromophore in bacteriorhodopsin, the chromophore in GFP has [31] an extended conjugated π-system. The positive charge in the Schiff base was stabilized by delocalization, which suggested the possibility of a similar stabilization in GFP. Several attempts were made to transfer a proton from the guanidinium group of Arg96 to the oxygen on the imidazole ring, in the hope that the resulting cationic charge on the chromophore would be stabilized in a similar manner. These all failed. This negative result could be regarded as confirmation that the original 1994 description [31] of the neutral chromophore was correct.

The secondary structures of both bacteriorhodopsin and GFP are held together by a large number of hydrogen bonds. As a result, both of these structures are relatively rigid.

In contrast to these two proteins, the structure of barnase involves two α-helices and a multi-strand antiparallel β-sheet, as well as a β-hairpin bend, and several intrinsically disordered regions, and, as such, provided a useful test for the ability of PM6-ORG to reproduce the experimentally-observed geometry. Given that the PM6-ORG RMSD of the barnase backbone, 0.86 Å ( Table 6), is slightly lower than the average, 0.95 Å (Table 8), the inference can be made that the predictive power of PM6-ORG to reproduce the geometry of proteins is not significantly impaired by disorder in the protein geometry.

The smallest protein examined, crambin, with only 46 residues, contains two short α-helices; the rest of the structure is disordered. Because of these features crambin has been used extensively as a test case for experimental work and for computational modeling, and for the same reasons was chosen as a test case in the early stages of this work. Only one caveat is made regarding its usefulness: because RMSD tends to increase with system size, and because crambin was the smallest protein, the RMSD, at 0.80 Å, is, to a degree, artificially small. Nevertheless, there is no indication that this would compromise the significance of the results.

Two proteases, chymotrypsin and 3CLpro were selected. To catalyze the peptide bond hydrolysis, chymotrypsin uses a catalytic triad Ser195, His57, and Asp102, and 3CLpro uses the dyad His41 and Cys145. In both enzymes the binding site is adjacent to the reaction site. Simulation of the catalytic mechanism of chymotrypsin has already been modeled [1], and, as mentioned earlier, is not controversial.

Of topical interest is the binding site of 3CLpro. Examination of the optimized PM6-ORG geometry of this site indicated that it was reproduced with useful accuracy, and tests to establish that it had the capability of binding known ligands are currently underway; preliminary results suggest that their results are encouraging.

### Proteins containing other elements

#### Sodium

In a biochemical environment, sodium atoms are invariably ionic. That is, they do not form covalent bonds. Thus, modeling their behavior presents problems; but, a simple test would be to model the behavior of sodium ions in a channel. In such an environment the ions could be expected to be able to migrate along the channel in response to an electrophoretic force: that is, there should be little resistance to motion of the ions along the channel in response to an electric field.

**Fig. 4:**
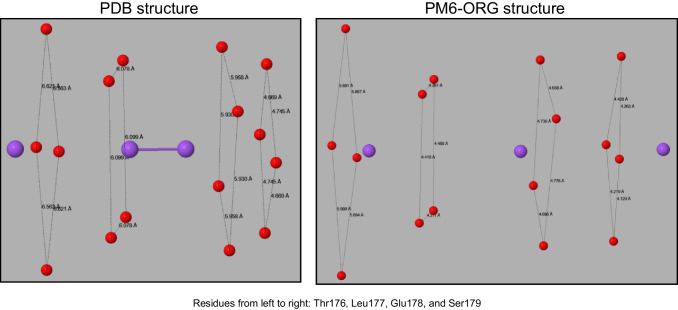
Sodium ion channel showing oxygen atoms nearest to the center of the channel

In the X-ray structure of the prokaryotic sodium channel protein 4CBC, four chains, A, B, C, and D, form the channel, the sides of which consist of oxygen atoms from the residues Thr176, Leu177, Glu178, and Ser179 of each chain. One oxygen atom from each residue contributes to the formation of a square, and each of these four squares is perpendicular to the axis of the channel. Three sodium ions are positioned on the axis, with two of them separated by 2.2 Å, as shown in Figure 4.

Geometry optimization resulted in the structure of the channel being conserved, albeit the width of the channel decreased about 12%, and the sodium ions migrated along the axis of the channel. Migration of this type would normally be regarded as an error, but in this system it is evidence that the computational model predicts that there would be little resistance to motion of sodium ions along the axis.

#### Magnesium

In biochemical systems, magnesium atoms occur in two important environments. One is at the center of a porphyrin ring, as in chlorophyll, the other is at the center of a set of oxygen atoms, normally six arranged in an approximately octahedral coordination.

In the PDB entry for peridinin-chlorophyll, entry 2X20, the magnesium atom forms bonds that range in length from 2.01 to 2.07 Å with the four nitrogen atoms of the porphyrin ring, and a fifth, non-covalent bond, of length 2.21 Å, with the oxygen atom of a water molecule located above the ring; for these quantities, PM6-ORG predicts the Mg-N bonds to be 2.06 – 2.15 Å long, and the Mg-O bond to have a length of 1.99 Å.

In the PDB structure of magnesium–loaded ALG-2, a magnesium atom is in an approximately octahedral environment of oxygen atoms, with five of the Mg-O distances ranging from 2.005 – 2.284 Å, and one at 2.858 Å. PM6-ORG predicts a similar environment, with five of the Mg-O distances ranging from 1.920 – 2.218 Å, and one at 3.459 Å.

#### Potassium

Potassium, like sodium, is invariably ionized, forming no covalent bonds, and therefore the only property of interest is its ability to migrate through a protein. In the X-ray structure of the KcsA potassium channel protein, PDB ID: 1JVM, the potassium ions were replaced by rubidium ions [32]. For the purpose of this work, these ions were replaced by the original, slightly smaller, potassium ions. The tunnel in the filter contains three K^+^ ions, a tetrabutylammonium cation, and one water molecule. All these species were retained in the modeling.

In the X-ray structure of 1JVM, two of the potassium ions are located at the center of a slightly distorted square antiprism of oxygen atoms, and the third ion is at the center of a slightly distorted tetragonal prism. After the geometry was optimized these structures were still present, but were significantly distorted, with the K – O distances ranging from 2.55 to 3.88 Å, whereas, in the X-ray structure, they ranged from 2.90 – 3.36 Å.

#### Calcium

The largest error in the predicted geometry of 1UOW involved one of the carboxylate oxygen atoms on the highly-conserved [33] Asp309 bonding to calcium. In the PM6-ORG geometry, this distance was 2.06 Å, while in the PDB geometry the smallest Ca – O separation was 2.41 Å. There is evidence of a significantly larger covalent interaction in the PM6-ORG geometry, in that the atomic partial charge on Ca bound to Asp309 was +1.24 in the PDB geometry, whereas in the PM6-ORG geometry this decreased to +1.05.

#### Iron

**Fig. 5:**
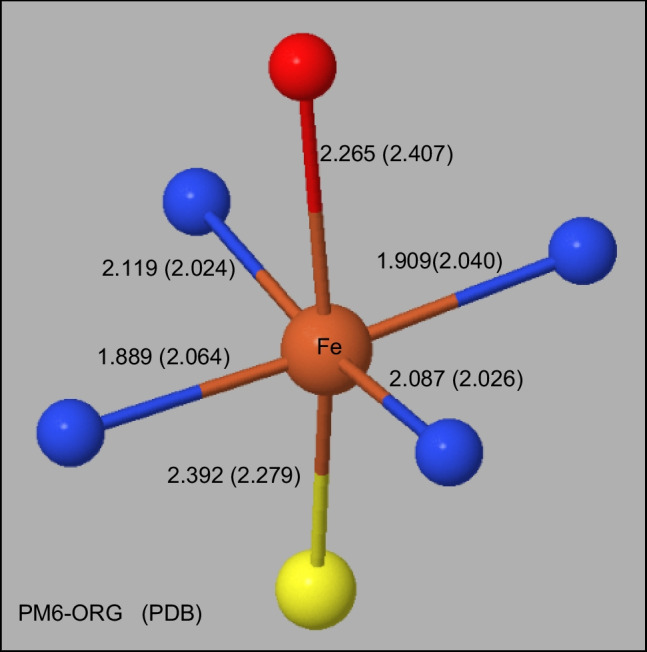
Environment of Iron in P450 Distances in Å

Two common forms involving a heme ring system were modeled. In one form, found in cytochrome P450, the iron atom is at the center of a porphyrin ring system, and covalently bonds to Cys332 at Sγ, and non-covalently binds to O3 of Gol402. A comparison of the X-ray and calculated environment of the iron atom is shown in Figure 5. This system, PDB ID: 7TTP, with 345 residues and 217 water molecules, was one of the larger systems studied.

The other form, found in human hemoglobin is tetrameric. The X-ray structure of this protein, PDB ID: 5WOH, contains 566 residues and is very large. For computational convenience, only the first subunit consisting of 137 residues and 197 water molecules was modeled; in this subunit, iron forms a covalent bond with His87 at Nε2 and a non-covalent bond with the oxygen of H_2_O312, in addition to the standard porphyrin ring system. The environment of the iron atom is similar to that in P450, except that in P450 there is a Fe-S covalent bond, in hemoglobin there is a Fe-N covalent bond. PM6-ORG predicts this bond to be1.993 Å long, versus the PDB value of 2.135 Å.

#### Cobalt

**Fig. 6:**
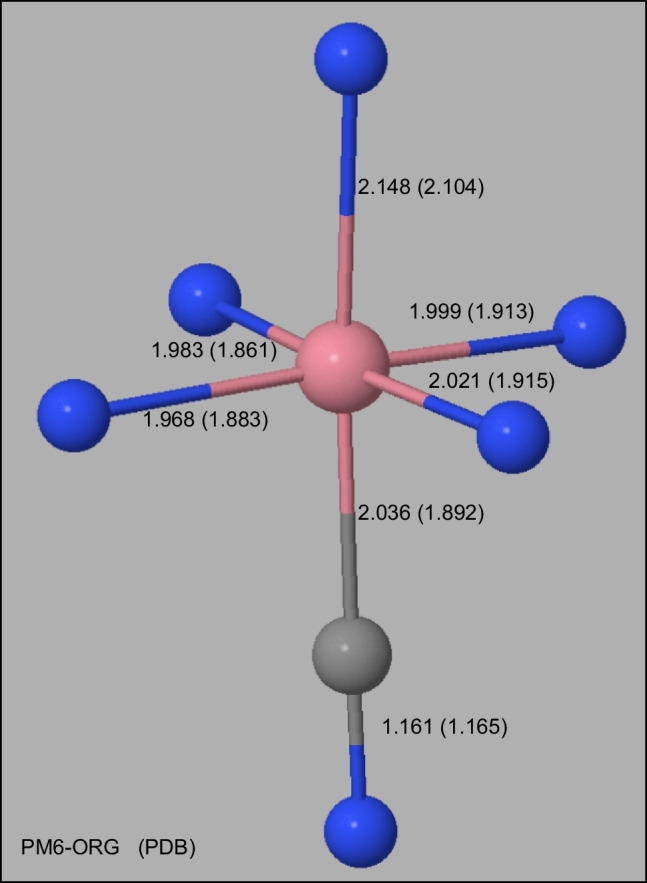
Environment of Cobalt in Transcobalamin Distances in Å

In PDB ID: 2V3N, the cobalt atom is octahedrally coordinated to the four nitrogen atoms of a corrin ring, a nitrogen atom of a dimethylbenzimidazole group, and a carbon atom of a cyano group. A comparison of its X-ray and calculated environment is shown in Figure 6.

#### Zinc

In the zinc finger, the average of the six Zn-N distances was predicted to be 2.054 Å, identical to that in the X-ray geometry, and the average Zn-S distance was 2.311 versus the X-ray value of 2.298 Å.

The various bond-lengths in zinc endoprotease are shown in Table 9.

**Table 9:** Bond lengths from Zinc in Zinc endoprotease (Å)

Bond	PM6-ORG	PDB
Zn-O (H_2_O202)	1.998	1.933
Zn-O (Asp93)	1.894	1.948
Zn-N (His87	1.984	2.014
Zn-N (His83)	1.975	2.006

In biochemical systems, zinc invariably occurs as Zn^II^, and almost always adopts a tetrahedral coordination, bonding to a combination of nitrogen, oxygen, and sulfur atoms, and therefore modeling its chemistry is relatively uncomplicated.

#### Selenium

In proteins, selenium occurs most often as selenomethionine, the selenium analogue of methionine, where it forms two covalent bonds with carbon atoms. The average of the Se-C bond lengths in Rab6, PDB ID: 1D5C, and adenylyltransferase, PDB ID: 1O6B, was 2.000 Å, slightly longer than the average of the X-ray structures of 1.921 Å. The C-Se-C angles averaged 97.4^0^ compared to the X-ray value of 100.0^0^.

### Comparison of physics and computational chemistry results

There are fundamental differences in the ways that experimental physics methods and computational chemistry model proteins.

In the physics approach, information from experimental samples is used to generate three-dimensional models of proteins and any associated small molecules. The general objective of these methods is to produce structures that are as similar as possible to those that would exist *in vivo* so that they can be a source of information for use in biochemistry. The Protein Data Bank contains a large curated collection of experimentally-determined structures, together with validation reports on their quality. These usually include the results of a Molprobity analysis of the structure; analyses that have high clashscores normally indicate lower-quality structures.

Experimentally-determined protein structures provide an invaluable insight into the biochemical processes that occur in enzymes. For example, examination of the structure of chymotrypsin allowed the complicated catalytic mechanism used in the hydrolysis of a peptide bond to be elucidated, including the discovery of the significance of structures such as the catalytic triad and the oxyanion hole and their role in reducing the activation barrier.

On the other hand, the objective of computational chemistry methods is to model physical and chemical phenomena that occur in protein chemistry. Two of the potentially most important applications involve modeling the binding of ligands to various sites in enzymes, and modeling the mechanisms used by enzymes to catalyze reactions.

Another difference is that computational chemistry is dominated by energy considerations: calculation of the heats of formation and their derivatives with respect to geometry is essential for locating stable intermediates and transition states. Calculated heats of formation of reactants, transition states, intermediates, and products for a reaction mechanism can not only be used in understanding the mechanism, they provide considerably more insight than can be obtained when only the static experimental geometries are used. For example, a quantitative estimate can be made [1] of the relative importance of the oxyanion hole and the catalytic triad in lowering the activation barrier in chymotrypsin. Another example, also in chymotrypsin, concerns the role of the catalytic triad residue Asp102. In the literature, the general consensus has been that at all stages in the mechanism this particular residue is ionized, yet the suggestion has also been made [34] that at one point in the catalytic cycle it might be neutral. When the mechanism was modeled, the results of the computational method agreed with the consensus, predicting that in the lowest energy path Asp102 was always ionized, and that neutralizing it at any point inevitably resulted in an increase in energy.

Correcting the large clashscore error in PM7 and PM6-D3H4 required only the addition of a very simple function that represented the missing vdW repulsion between well-separated atoms. As mentioned earlier, from a practical perspective the precise form of the repulsion function was unimportant. A single-sided Gaussian was used in this work, but any similar alternative, such as the hyperbolic tangent function, would also have been perfectly acceptable. Adding this correction resulted in a large reduction in clashscores without introducing any significant errors. The significance of this improvement is that existing semiempirical chemistry models lacked a vdW repulsion term, and consequently the predicted protein geometries had large clashscores, but, when the repulsion term was added, the fault was corrected and the clashscores decreased dramatically.

### Importance of appropriate weightings

The first step in converting a reference datum into a form suitable for use in optimizing parameters is to render it dimensionless. In this work, data representing energies were assigned weighting factors that depended on the energy range involved, from about 1 up to 100/(kcal mol^-1^).

In addition to the weighting factors depending on the types of reference data used in the training set, weighting factors can also be used in altering the focus or objective of the method. In the present work, the aim was to reduce the clashscores and to improve the accuracy of prediction of intermolecular interactions of the type found in ligands non-covalently bound to proteins. The results of using this particular focus can be seen in Table 3 and Table 8. If a different focus were to be desired, for example, to increase the accuracy of intermolecular interactions, then the appropriate weighting factors would need to be changed: i.e., reduce the factors for clashscores or increase the factors for intermolecular interactions or both, then the parameter set re-optimized. To assist in such a parameterization project, all files that were used in generating PM6-ORG are made available in the Supplementary Material. Operations of this type are straightforward, and would allow a method tailored to any specific need to be developed.

### Accuracy of PM6-ORG

The following summary of the features of the new method can be used in deciding its applicability to any specific protein system.

Overall, clashscores improved in going from an average of 32.4 for PM6-D3H4 and 28.5 for PM7 to 4.8 for PM6-ORG.

The average RMS errors in the geometry of protein backbones increased by 14% relative to PM6-D3H4, but were still very slightly smaller than those in PM7. This increase was accompanied by an increase of 0.6% in the difference of the volumes of the proteins.

Two proteins optimized using PM6-D3H4 had severe faults. In transcobalamin, a spurious covalent bond formed between Cys98 and H2O2031, and in zinc endoprotease a spurious bond formed between Cys112 and Arg79. Neither of these faults was present in the PM6-ORG optimized structures, nor were any new faults found.

### Limitations

Most of the limitations in the applicability of PM6-ORG to modeling proteins are caused by the underlying software limitations of the MOPAC program. Because of their size, solving the SCF equations for proteins always requires the MOZYME procedure to be used. In its current form, MOZYME is limited to closed-shell systems, so open-shell systems of the type encountered in free radical biochemistry and photochemistry, e.g., photosynthetic pathways and other proton pumps, cannot be modeled.

Another class of systems that could cause problems involves heterocycles with transition metal ions in their center, such as corrin with Co^III^ as in transcobalamin, and porphyrin with Fe^II^ in P450. In their ground states the geometries of these systems can be modeled using MOZYME, as shown in 2V3N and 7TTP, but modeling electronic phenomena of the type that occur in reaction mechanisms when the oxidation state of a metal atom changes would not be possible.

About 7,000 atoms is the practical upper limit of the size of system that can be modeled. The most time-consuming step in a modeling study is the initial geometry optimization. For a system of 7,000 atoms and using a 3GHz computer this would require about one to two CPU weeks. This limit can be avoided for proteins in which allosteric behavior is not important. In such systems, all chemical effects on binding sites and active sites caused by distant atoms can be ignored, so that the system could be trimmed down to only include atoms within about 12 Ångstroms of any atom of interest. The resulting system would then be in the 1,000 – 2,000 atom range and simulations would run much faster. In practice, initial geometry optimizations of trimmed systems required only one or two days and subsequent optimizations would run in just a few hours.

### Use of PM6-ORG for investigating protein chemistry

Although the addition of the vdW repulsion term to the computational method improved the clashscores for proteins, in order to carry out meaningful simulations other criteria relating to the computational model must also be satisfied. These criteria all involve issues relating to chemical behavior. Resolving some of these might present difficulties, so the following suggestions are provided in the hope that they might prove useful.

#### Preparing proteins

Before attempting to model protein behavior it is essential that several steps must be carried out. The first, and by far the most important of these, is that the model should be as realistic as possible.

A good starting point for this is to hydrogenate the experimentally-generated geometry of a protein together with any associated small molecules such as water and, if present, a ligand. Three steps are involved.

First, add hydrogen atoms to neutralize all sites in the protein except for non-covalently bound atoms that would normally be ionized, such as Ca^+2^, Na^+^, K^+^ and Cl^-^. Second, ionize all ionizable residues. Third, add and delete hydrogen atoms to ensure that the starting model structure is correctly ionized.

To simulate the *in vivo* environment of a protein, the use of implicit solvation is essential. For this, the COSMO model is ideal.

Generating a self-consistent field for macromolecules is best done using the MOZYME localized molecular orbital approach. MOZYME begins by generating a Lewis structure for the entire system. One result of this process is the generation of a list of all ionized sites, information which is extremely useful when checking for errors in hydrogenation and bonding. The LMO’s are then constructed and used in solving the SCF equations. An incidental beneficial result of using Lewis structures as the starting LMO’s is that a common problem when working with proteins, ensuring that the net charge is correct, is eliminated.

#### Problematic protein geometries

Experimental geometries that have low clashscores typically do not present any problems, but geometries with high clashscores can present problems with hydrogenation and with solving the SCF equations. The default method in MOPAC for hydrogenating systems relies on the topology of the system, but large clashes can alter the topology to such a degree that the resulting system becomes unrecognizable. By using keywords to selectively edit the topology, a chemically-sensible structure can be generated and hydrogenated. Because the starting LMO’s used by MOZYME depend on the topology, an incorrect topology could cause MOZYME to generate an incorrect SCF. This particular fault can also be avoided by using the same keyword that was used to edit the topology for hydrogenation to edit the topology used in constructing the starting LMO’s.

**Fig. 7:**
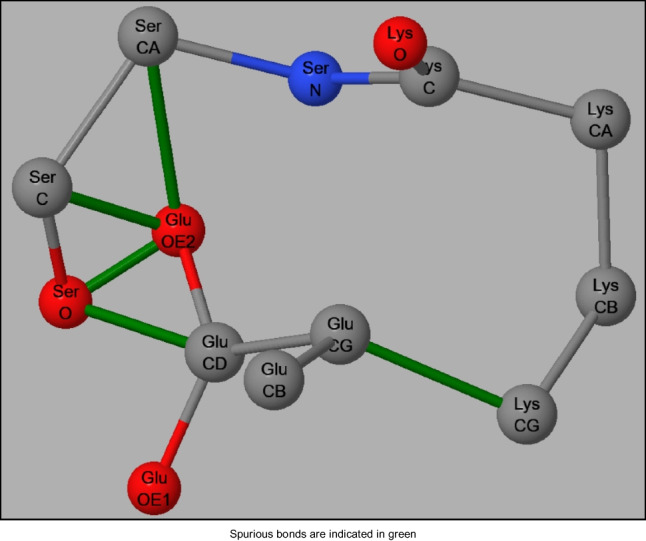
Topology of residues Glu16, Lys19 and Ser20 in Chain D in 1PY4

An example of a non-standard topology can be seen in PDB ID: 1PY4, a protein of 388 residues. The X-ray structure of 1PY4 has a resolution of 2.90 Å and a wwPDB validation clashscore of 104, and consists of four chains, A, B, C, and D. Chain D is unusual in that the carboxylate group of Glu16 is in close proximity to the four backbone atoms of Ser20, and Cγ on Glu16 is close enough to Cγ on Lys19, 1.73 Å, to give rise to the incorrect assumption that these atoms are covalently bonded together. The orientation of these residues is shown in Figure 7. This disorder was indicated by the reported fractional occupation of the atoms in these residues of 0.01.

Five topologic bonding errors were generated by MOPAC and are shown in green. By selectively deleting these connections, the correct topology would be revealed and the system could then be correctly hydrogenated. The same set of deletions that was used in hydrogenation would also be used to correct the topology in preparing the LMO’s for MOZYME. When the geometry was optimized using PM6-ORG the calculated clashscore dropped from 104.50 to 1.58. As would be expected from a system with such a high clashscore, the RMSD, 1.564 Å, was unusually large.

#### Geometry optimization

The next step would be to optimize the geometry of the system and compare the results with the experimental geometry. Known faults in the calculated geometry, such as systematic backbone motion, can safely be ignored; instead, attention should be focused on the local environment of sites of interest. In general, the geometries of reaction and binding sites are quite strongly conserved [35], so any significant distortion from the experimental geometry would be a cause for concern. Whenever that occurs, further simulations should be postponed until the cause of the distortion is found, and, if necessary, a correction made. Two of the most common causes are incorrect ionization of residues and missing water molecules. Identifying incorrectly ionized sites is straightforward – the geometric change caused by the incorrect presence or absence of a charged site is normally obvious. Deciding whether a water molecule is missing is more problematic. In one case [2], the experimental structure contained two copies, “A” and “B,” of the protein being studied. Copy “A” was selected as the starting system. When the optimized geometry was examined, an unusually large distortion was found in the binding site. Examination of the “B” structure showed that there was a water molecule at that site that was missing in the “A” structure. When the missing water molecule was added to the “A” structure, the distortion vanished.

In side-chains of the residues glutamine and asparagine, where incorrect assignment of oxygen and nitrogen can occur, and histidine, where flipping is possible, errors in conformer orientation can usually be detected by the large distortions that appear in the optimized conformer, or by obviously incorrect or missing hydrogen bonds. More exotic errors, such as a Mg^2+^ ion being mistaken for a water molecule, can also be identified by the relatively large distortions that occur in water clusters.

## Summary

By using a reference data set tailored to focus on systems relevant to protein chemistry and adding computed benchmark reference data for repulsive interactions, as well as adding a small correction to account for long-range repulsions, an improved semiempirical method for modeling proteins has been developed. Four faults in its parent method, PM6-D3H4, were corrected: the result of the addition of D3H4 to PM6 was a large increase in average errors in heats of formation; a known fault in PM6 produced spurious sulfur-oxygen and sulfur-nitrogen covalent bonds; a tendency to form too many hydrogen bonds; and, in proteins, the large number of clashes detected using Molprobity. Computed properties of 21 proteins were significantly improved, and an examination of sites of interest indicated that the new method, named PM6-ORG, should be more suitable than the preceding methods in MOPAC for modeling enzyme mechanisms and protein-ligand interactions.

## Data Availability

All software, training and survey sets, and results cited in this study are available at HTTP://openmopac.net/PM6-ORG.
